# Legal Regulation of Medical Practice

**Published:** 1869-05

**Authors:** 


### Editorial Department.

### Legal Regulation of Medical Practice.

Several of the States have, within the last year, passed laws "Regulating the Practice of Medicine," the main feature of which in all cases, has been to exclude from practice all who have not diplomas from incorporated medical colleges or other legal boards. Having been in practice for a specified number of years constitutes an exception to the general rule, but in their future operation these laws are designed to effect this end. So far as the medical profession is concerned, there appears to be no great gain, as we are yet able to see, but no doubt the general feeling is that of satisfaction; doubtless these laws have been framed and urged by men who have had in view the advancement of medical science as well as the good of mankind. The regular profession require no protection from unlicensed, uneducated or itinerant impostors, and we presume that it was no part of object in "regulating the practice of medicine" to extend any such aid. There are in all these States some physicians who have been engaged in practice who have no diplomas, and yet are not less capable, intelligent or trustworthy than their neighbors, who hold diplomas and are not "regulated" by the law. These must now complete their terms of study and receive the necessary credentials. But diplomas from incorporated colleges, and licenses from legal boards are easily obtained, and these laws all fail to afford the people the protection they so much need.

If people are too ignorant to be able to provide for themselves suitable and safe attendance when sick, their rulers and guides should certainly afford all possible relief, but when this is the case with the law-makers themselves, there can be no great good made to grow out of their interference. It has always appeared to us that but one plan offers any permanent relief. We must in some degree educate the masses; must afford the people an insight into the true principles of rational medicine; must ourselves reject all pretence not founded in fact, and claim for our art that which and nothing more than rightfully belongs to it; must talk correctly about the nature of disease and the true value of medicine, hoping in time to educate a people who will despise and shun pretension and quackery wherever exhibited.

We constantly know of families and individuals who have a very strange relish for being imposed upon; who go the rounds of quacks and patented nostrums with an obstinacy becoming thicker skins and longer ears, and we often think, and sometimes say, if they have any real ills, better to die and get out of the world as soon as possible, giving room for a more sensible race. It is not to be disguised that many of our "good families"—people who read, write, speak, and in other matters think correctly, are too ignorant of themselves and of all medical matters to be capable of self-care when sick. We build asylums for the insane and force those who thus suffer, within their walls, and we have sometimes feared that medical delusion would gain such extensive influence that sick people would in like manner require forced restraint and care.

If the States demand immunity from the ignorant, designing, graceless scamps who call themselves doctors, and will imprison all who practice villainies under such signs, as counterfeiters and thieves are imprisoned, and will rigidly require all graduates in medicine, who are allowed the care of the sick, to be in some degree educated men, then we may truly believe that the government is wiser than the people.

We have never looked to the law as capable of affording any relief from the wide-spread influence of ignorance and superstition, but if the legislatures which have required a "diploma," will now regulate also the "diploma," the advance in common sensibility will be astonishing. The remedy will be complete if each State will exclude from practice those who are *uneducated.* We have nothing more to desire than that all graduates in medicine be educated men. As it now stands, a few months in any of the *progressive (?)* schools, affords diploma from incorporated (?) college; but, O! what a burlesque upon the diploma which truly represents a medical education. To this point we most respectfully call the attention of law-makers, and hope they will add the finishing clause of an exclusive examining board, independent of all schools of medicine. But, then, only think of an *Independent Examining Board,* appointed by a Governor, or elected by a legislative body; we think it would be likely to be truly *independent.*

